# Predictive models of insulin resistance derived from simple morphometric and biochemical indices related to obesity and the metabolic syndrome in baboons

**DOI:** 10.1186/1475-2840-8-22

**Published:** 2009-04-23

**Authors:** Alberto O Chavez, Amalia Gastaldelli, Rodolfo Guardado-Mendoza, Juan C Lopez-Alvarenga, M Michelle Leland, M Elizabeth Tejero, GianPio Sorice, Francesca Casiraghi, Alberto Davalli, Raúl A Bastarrachea, Anthony G Comuzzie, Ralph A DeFronzo, Franco Folli

**Affiliations:** 1 Department of Medicine, Diabetes Division, University of Texas Health Science Center at San Antonio, San Antonio, TX, USA; 2 Fondazione G. Monasterio and Institute of Clinical Physiology-CNR, Pisa, Italy; 3 Genetics Department, Southwest Foundation for Biomedical Research, San Antonio, TX, USA; 4 Lab Animal Resources. University of Texas Health Science Center at San Antonio, San Antonio, TX, USA; 5 Istituto Scientifico San Raffaele, Milan, Italy; 6 Department of Pharmacology, University of Texas Health Science Center at San Antonio, San Antonio, TX, USA

## Abstract

**Background:**

Non-human primates are valuable models for the study of insulin resistance and human obesity. In baboons, insulin sensitivity levels can be evaluated directly with the euglycemic clamp and is highly predicted by adiposity, metabolic markers of obesity and impaired glucose metabolism (i.e. percent body fat by DXA and HbA_1c_). However, a simple method to screen and identify obese insulin resistant baboons for inclusion in interventional studies is not available.

**Methods:**

We studied a population of twenty baboons with the euglycemic clamp technique to characterize a population of obese nondiabetic, insulin resistant baboons, and used a multivariate linear regression analysis (adjusted for gender) to test different predictive models of insulin sensitivity (insulin-stimulated glucose uptake = Rd) using abdominal circumference and fasting plasma insulin. Alternatively, we tested in a separate baboon population (n = 159), a simpler model based on body weight and fasting plasma glucose to predict the whole-body insulin sensitivity (Rd/SSPI) derived from the clamp.

**Results:**

In the first model, abdominal circumference explained 59% of total insulin mediated glucose uptake (Rd). A second model, which included fasting plasma insulin (log transformed) and abdominal circumference, explained 64% of Rd. Finally, the model using body weight and fasting plasma glucose explained 51% of Rd/SSPI. Interestingly, we found that percent body fat was directly correlated with the adipocyte insulin resistance index (r = 0.755, p < 0.0001).

**Conclusion:**

In baboons, simple morphometric measurements of adiposity/obesity, (i.e. abdominal circumference), plus baseline markers of glucose/lipid metabolism, (i.e. fasting plasma glucose and insulin) provide a feasible method to screen and identify overweight/obese insulin resistant baboons for inclusion in interventional studies aimed to study human obesity, insulin resistance and type 2 diabetes mellitus.

## Background

Non-human primates are valuable models for the study of human disease, because of their close genetic and physiologic similarity to man [1-3]. The baboon (*Papio hamadryas*) is a long lived primate with an average lifespan of ~25 years that can be maintained in controlled conditions for generations, in order to evaluate the interaction between genetic and environmental factors in the pathogenesis of complex diseases [1,4,5]. Baboons display biochemical and molecular characteristics of the insulin resistance (metabolic) syndrome and type 2 diabetes (T2DM), as they progress from a lean to an obese phenotype and during the normal process of aging [5-8]. Not surprisingly, these primates develop several pathologies similar to those in man and they have been widely used and established as models for osteoporosis, atherosclerosis and obesity [4,9-11]. Recently, using the hyperinsulinemic euglycemic clamp technique, we characterized the baboon as a model of insulin resistance and identified key biochemical and molecular defects in the insulin signaling cascade in some target tissues (skeletal muscle and adipose tissue) [12]. Our results demonstrated that insulin resistance is directly related to fat mass, with percent body fat measured by dual-energy X ray absorptiometry (DXA) being the best predictor for insulin resistance. However, in large scale studies, the feasibility of DXA scan as a screening method to identify insulin resistant baboons is limited, because it is laborious and expensive. At our Institution, as part of the routine animal husbandry, all baboons receive a biannual health check. While sedated for this health check, animals are also weighted and basic morphometric measurements are obtained along with a blood chemistry panel for metabolic profiling [4]. We sought to develop a simple screening strategy to identify lean insulin-sensitive and obese-insulin resistant baboons for inclusion in genetic, physiologic and pharmacologic studies of obesity and insulin resistance using morphometric and biochemical markers of adiposity and glucose metabolism. Our results demonstrate the value of simple morphometric (abdominal circumference) and metabolic measurements (fasting plasma glucose and insulin) to predict insulin sensitivity in this baboon non human primate model.

## Methods

### Study population and morphometrics

Twenty adult nondiabetic baboons (10 females and 10 males) with varying degrees of adiposity and insulin sensitivity comprised the original study population. For inclusion, sedated baboons were evaluated with morphometric measurements including weight, crown to heel length, BMI, abdominal circumference (measured with a flexible non-stretchable measuring tape at a level midway between the lower rib margin and iliac crest) and a biochemical panel during the course of a scheduled health check. Morphometrics and metabolic assessments were performed during the last scheduled health check for each baboon. The clinical and biochemical characteristics of this study population have been published elsewhere [12].

In addition, we studied a second group of baboons (n = 159) during a scheduled health check. Recorded measurements included body weight, fasting plasma glucose and insulin concentrations. Only baboons with a stable body weight over the previous six months, fasting plasma glucose <150 mg/dl and fasting plasma insulin concentrations <100 μU/ml were considered for inclusion. The purpose of this group was to validate a simpler predictive model using fasting plasma glucose and body weight derived from the first group (n = 20).

### Assessment of insulin sensitivity and calculations

Under general anesthesia, baboons received a 2-hour 60 mU/m^2^.min hyperinsulinemic euglycemic clamp after an overnight fast (~12 hour), as previously described [12,13]. During the steady state of the clamp (90–120 min) at the prevailing insulin concentrations, the skeletal muscle and adipose tissue are maximally stimulated, and demonstrated by an increased activation in key insulin signaling proteins starting at 30 min after insulin infusion [12]. Fasting plasma glucose (FPG) was measured by the glucose oxidase method (Beckman Glucose Analyzer 2, Beckman-Coulter, Fullerton, CA); plasma (FFA) free fatty acids were measured at baseline and by the end of the clamp using an enzymatic colorimetric assay (Wako Chemicals USA). Finally, fasting plasma insulin (FPI) concentration was determined using a commercial radioimmunoassay (Diagnostic Products, Los Angeles, CA) at baseline and at 10–15 minutes intervals throughout the euglycemic clamp. Steady state plasma insulin was calculated as the mean insulin concentrations from 90 to 120 min. Whole body insulin sensitivity was calculated as the mean glucose infusion rate during the steady state, reflecting the insulin-stimulated rate of glucose disposal (Rd). Alternatively, we calculated the whole body insulin sensitivity as the ratio between the Rd in milligrams of glucose per kg of lean body mass and SSPI (Rd/SSPI). Using the FPG and FPI, we also calculated the quantitative insulin sensitivity check index (QUICKI) as 1/log (FPI) + log (FPG) [14]. The adipocyte insulin resistance index (AIRI) that estimates the ability of adipocytes to suppress the rate of lipolysis in response to insulin was calculated as the fasting FFA concentrations multiplied by FPI (AIRI = FFA*FPI). Since baboons show a marked gender dimorphism in some morphometric and metabolic parameters, all analyses were adjusted for gender differences and variables with a non-normal distribution were log transformed prior to analysis.

### Development of predictive models of insulin sensitivity by morphometrics and metabolic assessment

In order to determine the predictive value of metabolic and morphometric measurements, we constructed two different models using a stepwise linear regression analysis, with Rd as the dependent variable. In the first model (Model 1), abdominal circumference, adjusted for gender, was the independent variable. In the second model (Model 2), we included abdominal circumference and FPI (log transformed) as the independent variables. Lastly, we created a model to predict Rd/SSPI (Model 3), by using fasting plasma glucose concentrations and total body weight as independent variables. We applied the formula for the prediction of Rd/SSPI to a second group of baboons (n = 159), in which body weight was stable over the previous 6 months, during a bi-annual scheduled health check that included measurements for body weight, fasting plasma glucose and insulin concentrations. Only baboons with a fasting plasma glucose <150 mg/dl and fasting plasma insulin concentrations <100 μU/ml were considered for the validation of this predictive analysis model.

Pearson's and/or Spearman partial correlations were calculated as appropriate. We used a step-wise approach to include significant correlations into the linear regression models. Statistical analyses were performed using Statview 5.0 (SAS Institute, Cary, NC) statistical package. A p value of < 0.05 was considered statistically significant.

## Results

### Insulin sensitivity and body fat composition measurements

In our study population, the Rd spanned a wide range of insulin sensitivity. Using a cut-off point for Rd <5 mg/kg.min to screen for insulin resistant primates we identified 10 insulin resistant baboons range and 10 insulin sensitive baboons. In baboons, similar to humans, there was a marked gender dimorphism in percent body fat, where females had significantly higher percent body fat compared to males (12 ± 2 vs. 6 ± 1.5%, p = 0.05).

### Morphometrics, markers of adiposity and insulin resistance in the adipocyte

In our study population, abdominal circumference correlated directly with the adipocyte insulin resistance index (r = 0.693, p < 0.02). When percent body fat content was measured using DXA, it also showed a strong correlation with the adipocyte insulin resistance index (R = 0.755, p < 0.001) (Table 1), but not with total and lean body mass.

**Table 1:** Correlations between the adipocyte insulin resistance index and morphometric markers of adiposity body fat composition and biochemical data.

**Variable**	**Correlation Coefficient**	**P value**
BMI (kg/m^2^)	0.731	0.001*

Abdominal Circumference (cm)	0.693	0.002*

Percent body fat (%)	0.889	0.001*

FPG (mg/dl)	0.261	0.313

HbA1c (%)	0.257	0.320

Suppression FFA_90–120 _(%)	0.153	0.558

* = two tailed level of significance < 0.05.

### Regression analysis and predictive models of insulin sensitivity

In a multivariate model, after adjusting for gender, the best determinants of insulin sensitivity were fasting plasma glucose (adjusted R^2 ^= 0.15), insulin concentrations (log transformed, adjusted R^2 ^= 0.32), total body fat (adjusted R^2 ^= 0.39), percent fat (adjusted R^2 ^= 0.46), and abdominal circumference (adjusted R^2 ^= 0.59).

**Figure 1:**
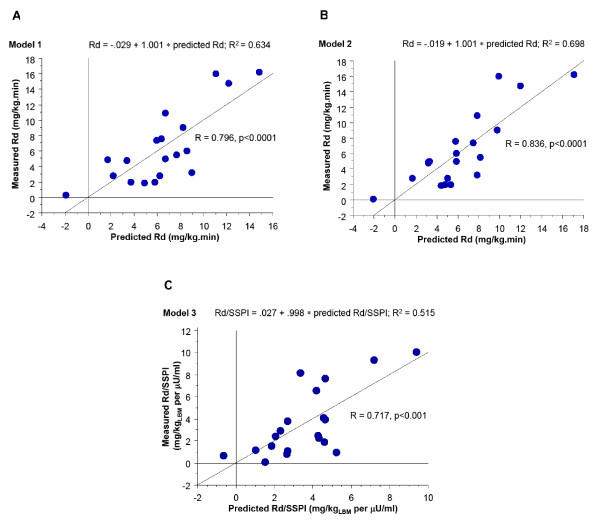
**Linear regression models to predict insulin resistance in adult nondiabetic baboons using (A) abdominal circumference (Model 1), (B) abdominal circumference + log FPI (Model 2), and (C) body weight + fasting plasma glucose (Model 3), as independent variables**. FPI = fasting plasma insulin.

In the first model (Model 1), we used abdominal circumference and gender to predict of tissue sensitivity to insulin (Figure 1-A, predicted Rd = -3.618*sex-0.376 * "Abdominal Circ" + 29.508, with sex = 1 for male, and 0 for females). The explained variability was 59%, with insulin sensitivity levels negatively correlated with abdominal circumference (partial R = -0.79, p < 0.0001) and gender (partial R = -0.55, p < 0.03). The regression analysis showed a high degree of correlation between the predicted and measured levels of insulin sensitivity (Figure 1A, R = 0.796, p < 0.0001). Since the increase in abdominal circumference is directly correlated with percent body fat and visceral obesity in baboons, this finding suggests that, similar to humans, a pattern of central adiposity predicts the presence of an insulin resistance metabolic syndrome in the baboon.

The addition of body fat measurements did not improve the model. On the other hand, the inclusion of log (FPI) in the second model (Model 2), overall improved its predictive power (adjusted R^2 ^= 0.64, predicted Rd = -4.894*sex-0.309 * "Abdominal Circ" -1.678* log (FPI) + 30.294, with sex = 1 for male, and 0 for females). However, despite the fact that abdominal circumference (partial R = -0.72, p = 0.001) and gender (partial R = -0.63, p = 0.007) were strong independent predictors of insulin sensitivity, the FPI was not independently correlated to Rd (partial R = -0.42, p = 0.09). The agreement between predicted and measured levels of insulin sensitivity is shown in Figure 1B (R = 0.836, p < 0.0001).

In Model 3, we considered weight and fasting plasma glucose as independent variables since these are the routine measurements collected during biannual health check. Model 3 also was able to predict well insulin sensitivity (Predicted Rd/SSPI = 17.18–0.305* Body Weight-0.056*FPG), explaining 51% of the insulin sensitivity variability as shown in Figure 1C (R = 0.717, p < 0.001).

**Figure 2:**
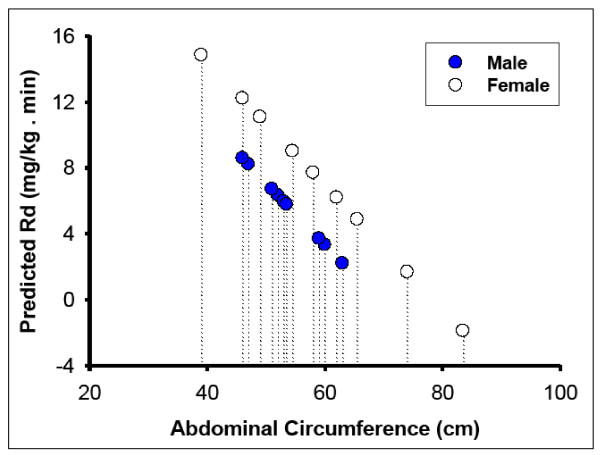
**Relationship between abdominal circumference (independent variable) and predicted rate of insulin-stimulated glucose disposal (Rd)**.

For simplicity, we generated a gender specific predictive scale, using the abdominal circumference model to predict insulin sensitivity (Figure 2). According to this model, an abdominal circumference >55 cm in males and >65 cm in females is highly predictive for insulin resistance in baboons.

### Body weight and fasting plasma glucose as predictors of insulin sensitivity in baboons

Although abdominal circumference was the strongest predictor of whole body insulin sensitivity, this measurement was not always readily available in health check records as a routine screening procedure. Therefore, we sought to validate Model 3 into a larger population in which abdominal circumferences were not available. Using this approach (Model 3 = body weight + FPG), we tested the predictive model in 159 baboons with a wide range of weight, fasting glucose and fasting insulin (Table 2). We stratified the animal population according to quartiles of predicted insulin sensitivity (Q1 = 38, Q2 = 41, Q3 = 39 and Q4 = 41). We found that the mean predicted Rd/SSPI was 8-fold higher in the insulin sensitive group compared to the insulin resistant quartile (Q1 = >8.4 vs. Q4 = <1.1 mg/kg_LBM _per μU/ml (plasma insulin), p < 0.001). For the whole population, the 25^th ^percentile was <2.906 vs. 75^th ^>7.252 mg/kg_LBM _per μU/ml (plasma insulin). The gender distribution showed gender dimorphism (males Rd/SSPI 25^th ^= 1.51 vs. 75^th ^= 4.537 mg/kg_LBM _per μU/ml (plasma insulin) and females Rd/SSPI 25^th ^= 4.66 vs. 75^th ^= 7.94 mg/kg_LBM _per μU/ml (plasma insulin).

**Table 2:** Clinical and metabolic characteristics of the baboon population (n = 159) used to apply and validate the ISI (Rd/SSPI) predictive model, distributed by quartiles.

**Variable**	**Q1** **(n = 38)**	**Q2** **(n = 41)**	**Q3** **(n = 39)**	**Q4** **(n = 41)**
Age (yrs)	20 ± 5	19.8 ± 4	18 ± 5	17.5 ± 5

Weight (kg)	24 ± 9	15 ± 2	26 ± 4	35 ± 6

FPG (mg/dl)	78 ± 12	88 ± 20	89 ± 19	97 ± 20

FPI (mU/ml)	32 ± 6	22 ± 5	37 ± 21	33 ± 6

FFA (mEq/L)	582 ± 60	587 ± 72	635 ± 56	687 ± 61

QUICKI	2.8 ± 1	2.7 ± 1	2.7 ± 1	2.8 ± 1

Values are expressed as mean ± SD. FPG = fasting plasma glucose, FPI = fasting plasma insulin, FFA = free fatty acids.

Q1 represents the most insulin sensitive quartile and Q4 represents the more insulin resistant quartile, FPG = fasting plasma glucose, FPI = fasting plasma insulin, FFA = free fatty acids, QUICKI = quantitative insulin sensitivity check index.

### Body weight and fasting plasma glucose are better predictors of peripheral insulin sensitivity in baboons

**Figure 3:**
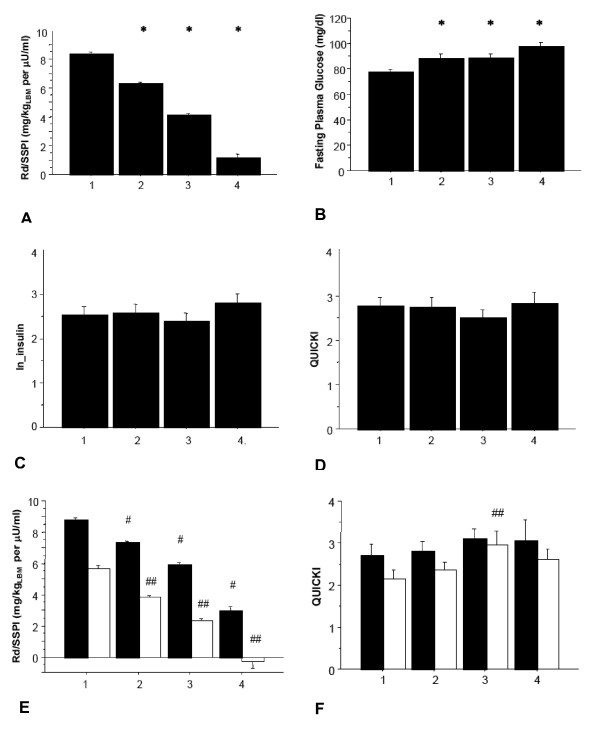
**Measurements of insulin sensitivity and glucose metabolism in a population of 159 baboons, divided by quartiles of insulin sensitivity**. (A) Rd/SSPI (B) FPG (C) log Insulin (D) QUICKI (E) Rd/SSPI adjusted for gender (F) QUICKI, adjusted for gender. Quartile 1 represents insulin sensitive baboons and Q4 insulin resistant baboons. White bar = male, black bar = female. * p < 0.05 vs. Q1 for whole group, # p < 0.05 vs. Q1 in females, ## p < 0.05 vs. Q1 in males.

Although there were clear cut differences in whole body insulin sensitivity (predicted Rd/SSPI) and a progressive increase in FPG from the lowest to the highest quartiles across the study population (Figures 3-A and 3-B), when we used surrogate indices of insulin sensitivity calculated during the fasting state (i.e. ln_FPI and QUICKI), no significant differences were found between groups (Figures 3-C and 3-D). Of note, the same results were obtained after gender specific comparison were performed (Figures 3-E and 3-F). This further supports the known limitation of FPI and QUICKI as surrogate measurements to differentiate peripheral (muscle) from central (liver) insulin resistance. Therefore, given their nature of steady fasting state measurements, FPI and QUICKI mainly reflect central (liver) insulin resistance [15,16].

## Discussion

In the present study, we evaluated the predictive value of simple markers of adiposity for the presence of insulin resistance (measured with the gold-standard euglycemic insulin clamp technique) in a population of adult nondiabetic baboons. Our results support the use of abdominal circumference as a simple and feasible measurement to predict insulin resistance in these non-human primates. Our findings are consistent with previous observations which demonstrated that baboons become insulin resistant as they develop a central obese phenotype [17]. In fact, it is well known that baboons develop early features of insulin resistance and metabolic syndrome along with a high body mass when they are exposed to unnatural nutritional and social environments (i.e. ingestion of high fat saturated human food waste in those living close to touristic facilities) compared to wild baboons subsisting mainly on forage [18]. During weight gain, baboons accumulate fat in the abdominal area (both visceral and subcutaneous) and the abdominal circumference is correlated with abdominal fat content and percent body fat. Although it is known that percent body fat correlates well with total body weight and abdominal circumference in both gender, there are not definitive established cut points for the diagnosis of obesity in baboons [4]. Therefore, in our study we have included baboons exhibiting a wide range of adiposity measured by abdominal circumference and percent body fat. Of note, when simple metabolic measurements and insulin sensitivity indices in the fasting state (such as FPG, FPI and QUICKI) are used in conjunction with abdominal circumference, the predictive power of the model increases and explains a very large portion (~70%) of the variation in insulin sensitivity.

Our study has several strengths: (i) the study population is a well characterized group of baboons from the morphometric, biochemical and molecular standpoint with clear cut differences between the lean, insulin sensitive and the obese, insulin resistant baboons; (ii) this nonhuman primate model mimics closely findings in humans that relate the presence of a central obese phenotype with impaired glucose metabolism and insulin resistance, indicating the presence of the insulin resistance (metabolic) syndrome equivalent in the baboon [19-23]. One potential weakness of our analyses is that we studied a relative small number of primates with the euglycemic clamp. However, we included baboons of both genders with a wide spectrum of insulin sensitivity, abdominal circumference and percent body fat. Moreover, we were able to validate a predictive model of insulin resistance based on total body weight and fasting glucose, which are useful surrogates to abdominal circumference when this is not readily available. Our findings are in agreement with other report and covered the range from the lean, insulin-sensitive to overweight/obese, insulin-resistant baboons [4,5]. Our findings also support the fact that insulin resistance is progressive, as baboons evolve from a lean to an overweight/obese phenotype. The insulin resistance is evident not only at the glucose uptake level in skeletal muscle, but also in the adipose tissue, as is demonstrated by the lack of suppression of FFA concentrations in the presence of high insulin levels in the baboons with the highest percent body fat, expressed as the adipocyte insulin resistance index. The estimation of adipocyte insulin resistance index provides some insight into the dysregulation of adipocyte biology present in obesity and the metabolic syndrome Therefore, the measurements of FFA and insulin could be of help to identify those baboons likely to have not only physical, but also metabolic and molecular features of the insulin resistant phenotype.

Although our results were obtained in baboons, they are likely to be applicable to other non-human primates, since the array of metabolic abnormalities associated with an obese phenotype is well documented across primate species [8,24-27]. Moreover, the use of morphometrics, specifically abdominal circumference is also very likely to predict insulin resistance when other methods different from the euglycemic clamp are used to quantitate insulin sensitivity (i.e. minimal model assessment from oral or IV glucose tolerance test), given the documented correlation between these techniques [28].

Our results are in agreement with several larger human cohorts demonstrating the predictive power of abdominal and trunk fat deposits to predict the presence of different traits of the insulin resistance metabolic syndrome and its co morbidities [21,29-31]. Although the use of abdominal circumference is a simple and feasible measurement that provides useful information about the insulin sensitivity and overall the metabolic status in baboons, it is not always readily available for screening purposes in primate studies. For this reason, we developed a model that included variables generally available and routinely collected as part of the semi-annual scheduled health checks at our institution. While the model considering body weight and fasting plasma glucose (Model 3) was not as powerful as abdominal circumference to explain the variability in insulin sensitivity, it was able to dissect clear cut differences in insulin sensitivity across the study group.

**Figure 4:**
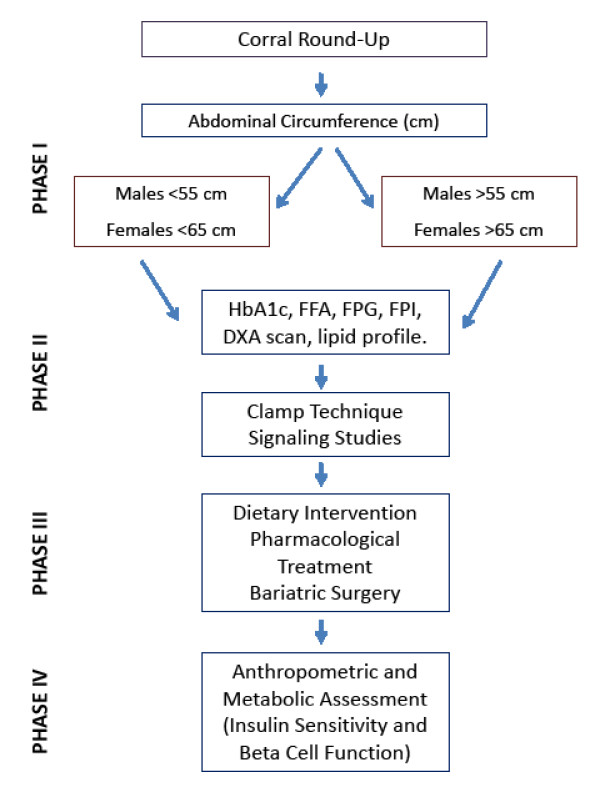
**Suggested screening procedure to identify insulin-sensitive and insulin-resistant baboons using abdominal circumference**.

These data further support the value of this baboon non-human primate as a model for the study of obesity and metabolic diseases in humans. Based on our results, we propose an algorithm for screenings of primates in order to identify those who are likely to present features of obesity or insulin resistance for inclusion in research studies (Figure 4). Since genetic, nutritional and environmental factors seem to play a complex integrated role in the pathogenesis of obesity and its associated traits, we strongly believe these findings could be applied to both wild and captive baboons.

In summary, abdominal circumference is a major determinant of insulin sensitivity in both male and female baboons, and its association with fasting measurements of glucose metabolism and insulin sensitivity, i.e. FPI and FPG, provide a useful instrument to screen primates to identify insulin sensitive and insulin resistant for inclusion in large scale protocols designed to examine the effects of dietary and pharmacological interventions in the study of obesity, insulin resistance and type 2 diabetes in humans.

## Competing interests

The authors declare that they have no competing interests.

## Authors' contributions

AOC conceived the study, performed the experiments, interpreted results and wrote the manuscript. AG conceived the study, analyze the data and participate in the writing of the manuscript. RGM collected and analyzed results. JCLA performed experiments and participated in the data analysis. MML conceived and performed the surgical part of experiments. MET, GS, FC, AD performed part of the experiments, draft and critically revised the manuscript. RAB and AGC conceived the study and participated in clamp experiments. RAD conceived the study and designed study experiments. FF conceived the study, designed study experiments, interpreted the results, participated in the writing and critically revised the manuscript.

## References

[B1] VandeBerg JL, Williams-Blangero S (1997). Advantages and limitations of nonhuman primates as animal models in genetic research on complex diseases. Journal of Medical Primatology.

[B2] Carlsson HE, Schapiro SJ, Farah I, Hau J (2004). Use of primates in research: a global overview. American Journal of Primatology.

[B3] Cox LA, Mahaney MC, Vandeberg JL, Rogers J (2006). A second-generation genetic linkage map of the baboon (Papio hamadryas) genome. Genomics.

[B4] Comuzzie AG, Cole SA, Martin L, Carey KD, Mahaney MC, Blangero J, VandeBerg JL (2003). The baboon as a nonhuman primate model for the study of the genetics of obesity. Obesity Research.

[B5] Cole SA, Martin LJ, Peebles KW, Leland MM, Rice K, VandeBerg JL, Blangero J, Comuzzie AG (2003). Genetics of leptin expression in baboons. International journal of obesity and related metabolic disorders: journal of the International Association for the Study of Obesity.

[B6] Guardado-Mendoza R, Davalli A, Chavez-Velazquez A, Comuzzie AG, Tejero ME, Lopez-Alvarenga JC, Bastarrachea R, Zuo P, Chang Z, Dick E (2008). Fasting Plasma Glucose (FPG) and HbA1c Predict Quantitatively Baboon Pancreatic Islet Amyloidosis (PIA): A novel Non-Human Primate Model of Beta Cell Failure in Type 2 Diabetes Mellitus (T2DM). Diabetes.

[B7] Hubbard GB, Steele KE, Davis KJ, Leland MM (2002). Spontaneous pancreatic islet amyloidosis in 40 baboons. Journal of Medical Primatology.

[B8] Tigno XT, Gerzanich G, Hansen BC (2004). Age-related changes in metabolic parameters of nonhuman primates. The journals of gerontology Series A, Biological sciences and medical sciences.

[B9] Rainwater DL, Kammerer CM, Cox LA, Rogers J, Carey KD, Dyke B, Mahaney MC, McGill HC, VandeBerg JL (2002). A major gene influences variation in large HDL particles and their response to diet in baboons. Atherosclerosis.

[B10] Harewood WJ, Gillin A, Hennessy A, Armistead J, Horvath JS, Tiller DJ (1999). Biochemistry and haematology values for the baboon (Papio hamadryas): the effects of sex, growth, development and age. Journal of Medical Primatology.

[B11] Rogers J, Hixson JE (1997). Baboons as an animal model for genetic studies of common human disease. Am J Hum Genet.

[B12] Chavez AO, Lopez-Alvarenga JC, Tejero ME, Triplitt C, Bastarrachea RA, Sriwijitkamol A, Tantiwong P, Voruganti VS, Musi N, Comuzzie AG (2008). Physiological and molecular determinants of insulin action in the baboon. Diabetes.

[B13] DeFronzo RA, Tobin JD, Andres R (1979). Glucose clamp technique: a method for quantifying insulin secretion and resistance. The American journal of physiology.

[B14] Katz A, Nambi SS, Mather K, Baron AD, Follmann DA, Sullivan G, Quon MJ (2000). Quantitative insulin sensitivity check index: a simple, accurate method for assessing insulin sensitivity in humans. Journal of Clinical Endocrinology and Metabolism.

[B15] Hirsso P, Rajala U, Laakso M, Hiltunen L, Harkonen P, Keinanen-Kiukaanniemi S (2005). Health-related quality of life and physical well-being among a 63-year-old cohort of women with androgenetic alopecia; a Finnish population-based study. Health Qual Life Outcomes.

[B16] Tripathy D, Almgren P, Tuomi T, Groop L (2004). Contribution of insulin-stimulated glucose uptake and basal hepatic insulin sensitivity to surrogate measures of insulin sensitivity. Diabetes care.

[B17] Cai G, Cole SA, Tejero ME, Proffitt JM, Freeland-Graves JH, Blangero J, Comuzzie AG (2004). Pleiotropic effects of genes for insulin resistance on adiposity in baboons. Obesity Research.

[B18] Kemnitz JW, Sapolsky RM, Altmann J, Muruthi P, Mott GE, Stefanick ML (2002). Effects of food availability on serum insulin and lipid concentrations in free-ranging baboons. Am J Primatol.

[B19] Janssen I, Katzmarzyk PT, Ross R (2004). Waist circumference and not body mass index explains obesity-related health risk. American Journal of Clinical Nutrition.

[B20] Kim SH, Abbasi F, Reaven GM (2004). Impact of degree of obesity on surrogate estimates of insulin resistance. Diabetes care.

[B21] Okosun IS, Liao Y, Rotimi CN, Prewitt TE, Cooper RS (2000). Abdominal adiposity and clustering of multiple metabolic syndrome in White, Black and Hispanic americans. Annals of Epidemiology.

[B22] Muscogiuri G, Chavez AO, Gastaldelli A, Perego L, Tripathy D, Saad MJ, Velloso L, Folli F (2008). The crosstalk between insulin and renin-angiotensin-aldosterone signaling systems and its effect on glucose metabolism and diabetes prevention. Curr Vasc Pharmacol.

[B23] Velloso LA, Folli F, Sun XJ, White MF, Saad MJ, Kahn CR (1996). Cross-talk between the insulin and angiotensin signaling systems. Proc Natl Acad Sci USA.

[B24] de Koning EJ, Bodkin NL, Hansen BC, Clark A (1993). Diabetes mellitus in Macaca mulatta monkeys is characterised by islet amyloidosis and reduction in beta-cell population. Diabetologia.

[B25] Hansen BC, Bodkin NL (1993). Primary prevention of diabetes mellitus by prevention of obesity in monkeys. Diabetes.

[B26] Wagner JE, Kavanagh K, Ward GM, Auerbach BJ, Harwood HJ, Kaplan JR (2006). Old world nonhuman primate models of type 2 diabetes mellitus. ILAR journal/National Research Council, Institute of Laboratory Animal Resources.

[B27] Tardif SD, Power ML, Ross CN, Rutherford JN, Layne-Colon DG, Paulik MA (2009). Characterization of Obese Phenotypes in a Small Nonhuman Primate, the Common Marmoset (Callithrix jacchus). Obesity (Silver Spring).

[B28] Dalla Man C, Yarasheski KE, Caumo A, Robertson H, Toffolo G, Polonsky KS, Cobelli C (2005). Insulin sensitivity by oral glucose minimal models: validation against clamp. American Journal of Physiology Endocrinology and Metabolism.

[B29] Bray GA, Jablonski KA, Fujimoto WY, Barrett-Connor E, Haffner S, Hanson RL, Hill JO, Hubbard V, Kriska A, Stamm E (2008). Relation of central adiposity and body mass index to the development of diabetes in the Diabetes Prevention Program. American Journal of Clinical Nutrition.

[B30] Nilsson G, Hedberg P, Jonason T, Lonnberg I, Tenerz A, Forberg R, Ohrvik J (2008). Waist circumference alone predicts insulin resistance as good as the metabolic syndrome in elderly women. Eur J Intern Med.

[B31] Pischon T, Boeing H, Hoffmann K, Bergmann M, Schulze MB, Overvad K, Schouw YT van der, Spencer E, Moons KG, Tjonneland A (2008). General and abdominal adiposity and risk of death in Europe. New England Journal of Medicine.

